# The effect of robenacoxib on the concentration of C-reactive protein in synovial fluid from dogs with osteoarthritis

**DOI:** 10.1186/1746-6148-9-42

**Published:** 2013-03-01

**Authors:** David Bennett, Peter David Eckersall, Mary Waterston, Veronica Marchetti, Alessandra Rota, Eilidh McCulloch, Silvia Sbrana

**Affiliations:** 1 School of Veterinary Medicine, College of Medical, Veterinary and Life Sciences, University of Glasgow, Bearsden Road, Bearsden, Glasgow G61 1QH, Scotland; 2 Institute of Infection, Immunity & Inflammation, College of Medical, Veterinary and Life Sciences, University of Glasgow, Bearsden Road, Bearsden, Glasgow G61 1QH, Scotland; 3 Department of Veterinary Clinics, Faculty of Veterinary Medicine, University of Pisa, Via Livornese Lato Monte, San Piero a Grado, Pisa 56010, Italy; 4 ReactivLab Ltd, Garscube Estate, Bearsden Rd, Glasgow G61 1QH, UK

**Keywords:** Stifle, Osteoarthritis, Cruciate disease, C-reactive protein, Synovial fluid, Robenacoxib

## Abstract

**Background:**

Robenacoxib is a novel and highly selective inhibitor of COX-2 in dogs and cats and because of its acidic nature is regarded as being tissue-selective. Thirty four dogs with stifle osteoarthritis secondary to failure of the cranial cruciate ligament were recruited into this study. Lameness, radiographic features, synovial cytology and C-reactive protein concentrations in serum and synovial fluid were assessed before and 28 days after commencing a course of Robenacoxib at a dose of 1 mg/kg SID.

**Results:**

There was a significant reduction in the lameness score (P < 0.01) and an increase in the radiographic score (P < 0.05) between pre- and post-treatment assessments. There was no difference between pre- (median 1.49 mg/l; Q1-Q3 0.56-4.24 mg/L) and post – (1.10 mg/L; 0.31-1.78 mg/L) treatment serum C-reactive protein levels although synovial fluid levels were significantly reduced (pre- : 0.44 mg/L; 0.23-1.62 mg/L; post- : 0.17 mg/L; 0.05-0.49 mg/L) (P < 0.05). There was no correlation between C-reactive protein concentrations in serum and matched synovial fluid samples.

**Conclusions:**

Robenacoxib proved effective in reducing lameness in dogs with failure of the cranial cruciate ligament and osteoarthritis of the stifle joint. The drug also reduced levels of C-reactive protein in the synovial fluid taken from the affected stifle joint. Robenacoxib appears to reduce articular inflammation as assessed by C-reactive protein which supports the concept that Robenacoxib is a tissue-selective non-steroidal anti-inflammatory drug.

## Background

Robenacoxib is a novel and highly selective inhibitor of COX-2 in cats and dogs [1,2] and is now available in several European countries for the treatment of articular pain and inflammation. Robenacoxib is an acidic NSAID and is highly bound to plasma proteins with the result that it concentrates in inflamed tissues such as an arthritic joint [3], thereby displaying the property of tissue selectivity [4]. The study described here was designed to show the effect of robenacoxib on the concentrations of blood and synovial fluid C-reactive protein (CRP), a recognized marker of inflammation. Robenacoxib was selected for this study because it has this capacity for tissue selectivity. In addition to assessing the effect of robenacoxib on CRP concentrations in blood and synovial fluid, its effect on lameness, radiographic changes and synovial fluid cytology in clinical cases of OA was investigated.

CRP is one of several acute-phase proteins (APPs) that can be used to assess the innate immune system’s systemic response to infection, inflammation or trauma [5-7]. These proteins are traditionally measured in serum and by definition their concentration increases by more than 25% in response to pro-inflammatory cytokines such as IL-6, IL-1 and TNFα which are released during the disease process. APPs are highly sensitive indicators of inflammation but unfortunately lack specificity. APPs are largely produced by the liver, although there is increasing evidence that other tissues can be a source [8].

APPs, particularly CRP and serum amyloid A (SAA) have been widely studied in human rheumatology as biomarkers of arthritic disease [9-13] and can be used to monitor response to therapy including NSAIDs [14]. Serum levels of SAA and CRP have been shown to correlate with disease activity in different types of inflammatory arthritis [15]. Buch et al. (2005) [16] examined the value of CRP as a predictor of response to therapy in patients with rheumatoid arthritis. The authors found that reduction in CRP levels following therapy was predictive of a clinical response within twelve to twenty-four weeks in over 50% of patients. Measurement of CRP is also a useful test in the diagnosis of infection after total knee arthroplasty [17] and septic arthritis in children [18]. Many studies have also examined concentrations of APPs in synovial fluid as well as serum of patients with inflammatory joint disease. In most cases serum levels are found to be higher than synovial fluid levels [13,19,20] although Kumon and co-workers [21] found that in some rheumatoid arthritis patients the synovial fluid levels of SAA exceeded the serum concentrations.

Increased concentrations of APPs in plasma and synovial fluid of osteoarthritic patients has also been demonstrated in man, although at lower levels compared to the inflammatory arthropathies [13]. More sensitive assays such as the ELISA are recommended for detecting the lower levels of APPs that are found in osteoarthritis [12,22]. Such assays have demonstrated modestly elevated blood levels of CRP in human osteoarthritic patients compared to aged-matched controls [23-26] and that increased levels of CRP are associated with disease progression [24,27,28] as well as with clinical severity [22]. It has also been reported that elevations in CRP may reflect events that may precede but ultimately lead to radiographic progression in osteoarthritis [25,28]. In addition elevated CRP has been found to be associated with the severity of pain in human OA [26] and a relationship between elevated blood levels of CRP, elevated synovial fluid levels of IL-6 and the presence of chronic synovial inflammation as assessed histologically has been described [22]. Pelletier et al. (2010) [29] reported that higher baseline levels of serum CRP in human patients are predictive of a greater risk of cartilage loss in OA and correlated with a worsening of symptoms including pain and function. CRP was also found to be a good predictor of the symptomatic response to treatment using NSAIDs. However, Kerkhof and colleagues (2010) [30] reported that although human knee OA was associated with 14% higher serum CRP levels, this association disappeared after adjustment for age and body mass index.

Very few studies examining the usefulness of APPs as biomarkers of canine arthritic disease have been published [31,32]. Although there are differences within species as to which APPs are most relevant to measure, CRP is accepted as the most useful in the dog, a situation similar to man. Caspi et al. [31] reported that serum CRP was found to be more elevated in dogs with active immune-based polyarthritis than in dogs with inactive disease or in affected dogs treated with corticosteroids, suggesting it might be a useful marker for the inflammatory arthropathies. Hurter and co-workers [33] examined the possible role of serum CRP as a marker of disease in OA by measuring serum levels in 29 clinically affected dogs. In this study all the measured values were within the normal limits, although they had a tendency to be higher than the control group. The authors concluded that serum CRP was not a useful clinical marker for canine OA. There have been no studies published on the measurement of CRP in the synovial fluid of dogs although one study did measure SAA levels in both serum and synovial fluid of dogs with inflammatory and non-inflammatory joint disease [34]. The numbers of dogs were too low to make any valid conclusions.

A recent collaborative study between the Universities of Glasgow and Pisa suggested that synovial fluid levels of CRP may be a useful marker of OA in the dog [35] and provided the stimulus for the present study. We studied 34 dogs with hindleg lameness caused by failure of the cranial cruciate ligament and secondary osteoarthritis. Blood and synovial fluid samples were collected when the dogs were presented and repeat samples were taken one month later after a course of Robenacoxib and CRP concentrations in the samples determined. The hypothesis was that treatment with the NSAID would significantly reduce the levels of synovial fluid CRP thus demonstrating its usefulness as a biomarker of articular inflammation.

## Results

**Figure 1:**
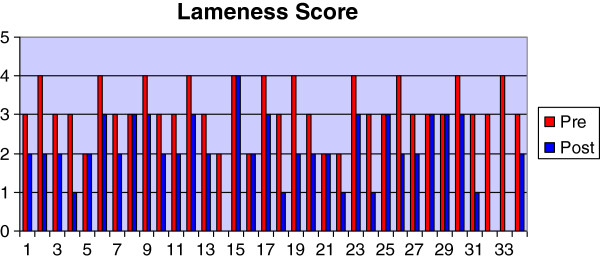
Graph showing pre- and post-treatment values for lameness score.

**Figure 2:**
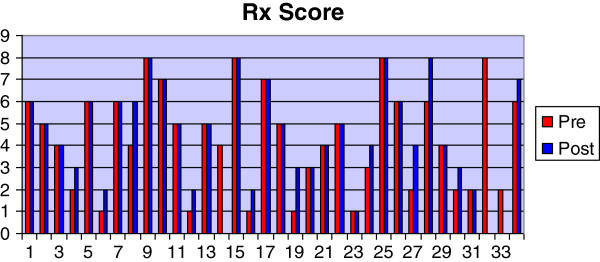
Graph showing pre- and post-treatment values for the radiographic score.

**Figure 3:**
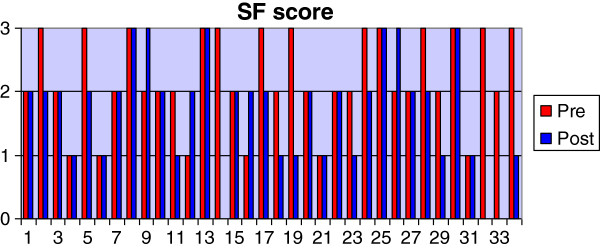
Graph showing pre- and post-treatment values for the synovial fluid score.

Most of the dogs were of the heavier breeds and crossbreeds with a mean body weight of 35.3 Kg and a range of between 19–60 Kg (Table 1). The most common pure-breeds were the Labrador Retriever (23.5%) and the Golden Retriever (11.8%) although cross-breeds accounted for 26.5% of the total. The mean age was 4.7 years with a range of 1–12 years. The mean duration of lameness was 11.1 weeks with a range between 1.4-34 weeks. The mean body condition score was 3.0 (ideal) with a range of between 2 (lean) and 5 (obese). No adverse effects relating to the NSAID were reported. Twenty-three of thirty-one dogs improved their lameness score after the course of Robenacoxib; by one grade in 16 dogs and by two grades in 7 dogs. The score stayed the same in 8 dogs (Figure 1). There was a significant reduction in lameness score between pre- (median = 3.0; Q1-Q3 = 3.0-4.0) and post- (2.0; 2.0-2.25) treatment assessments (P < 0.01). The radiographic score was unchanged in 20 of 31 dogs and was increased by one grade in 7 dogs and by two grades in 4 dogs (Figure 2). There was a significant difference between pre- (5.0; 2.0-6.0) and post- (5.0; 3.0-6.0) treatment radiographic scores (p < 0.05). The synovial fluid score was the same in 16 of 31 dogs, had decreased by one grade in 9 cases and by two grades in 2 cases. The score had increased by one grade in 4 cases (Figure 3). There was not a statistically significant difference between pre- (2.0, 1.7-3.0) and post- (2.0, 1.0-2.0) treatment synovial fluid scores although a tendency for a decrease was observed (p < 0.1).

**Table 1:** Summarising details of the 34 dogs with stifle osteoarthritis

**Case**	**Sex**	**Breed**	**Age**	**Weight**	**CCL**	**L. D.**	**P.T.**	**BCS**
1	M	Labrador R	48	36	L	45	0	3
2	M	Labrador R	50	36	R	20	0	3
3	F	Golden R	60	32	R	90	0	3
4	M	New Foundland	30	50	R	120	0	2
5	M	Golden R	60	30	R	60	0	3
6	F	Cross breed	48	26	L	20	0	2
7	F	Spinone	36	31	L	45	0	3
8	M	Bullmastiff	60	56	R	30	0	4
9	F	Cross breed	120	28	L	20	0	3
10	M	Pittbull	84	36	L	120	0	3
11	M	Pittbull	84	36	R	60	0	3
12	M	Labrador R	40	28	R	20	0	3
13	M	Labrador R	36	27	L	48	0	3
14	F	Beagle	96	20	R	72	0	4
15	F	Shar pei	48	28	R	30	0	3
16	F	Corso	12	54	L	120	0	2
17	M	Golden R	60	42	L	90	0	5
18	F	Cross breed	48	34	L	180	0	3
19	M	Cross breed	48	34	R	20	0	3
20	M	German Shepherd	72	24	L	30	0	4
21	F	Cross breed	60	32	R	90	0	4
22	M	Labrador R	24	36	R	180	0	3
23	F	Labrador R	36	43	L	10	0	3
24	F	Pittbull	96	40	R	30	0	4
25	M	Cross breed	84	38	L	240	0	3
26	F	Australian Shepherd	120	24	L	120	0	2
27	M	Corso	24	48	L	180	0	3
28	M	Great dane	30	60	R	90	0	3
29	M	Cross breed	48	35	L	180	0	4
30	F	Golden R	36	29	R	15	0	3
31	F	Labrador R	60	42	R	150	0	4
32	F	Cross breed	60	25	R	60	0	3
33	M	Cross breed	72	41	L	30	0	4
34	M	Labrador R	36	20	R	30	0	4

M: male; F: female; CCL: cranial cruciate ligament; L: left; R: right; L.D.: lameness duration; P.T.: previous treatment; BCS: body condition scoring. Age is given in months, weight in Kg.

### Serum CRP concentration

**Figure 4:**
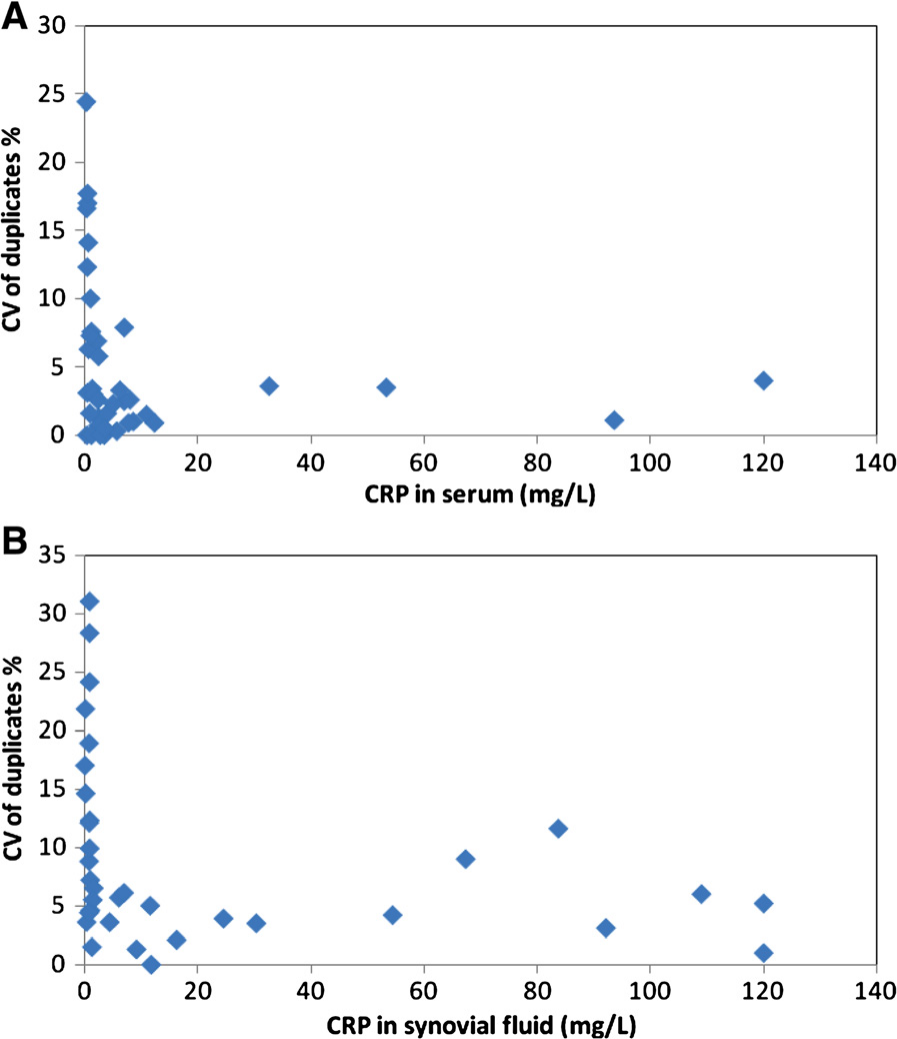
Precision profiles of CRP in (A) serum (n = 39) and (B) synovial fluid (n = 34) assayed in duplicate by ELISA.

**Figure 5:**
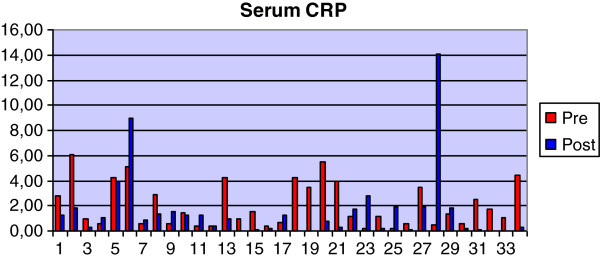
Graph showing pre- and post-treatment values for CRP concentration in serum.

The ELISA to determine the concentration of CRP in serum has been previously validated by Kjelgaard-Hansen and colleagues (2003) [36] and was confirmed in our study (detailed data not provided) (Figure 4). Fifteen of 24 dogs had a decreased concentration of CRP and 9 cases an increase after treatment (Figure 5). There was no significant difference between pre- (median 1.49 mg/l; Q1-Q3 0.56-4.24 mg/L) and post- (1.10 mg/l; 0.31-1.78 mg/L) treatment concentrations of serum CRP.

### Synovial fluid CRP concentration

**Figure 6:**
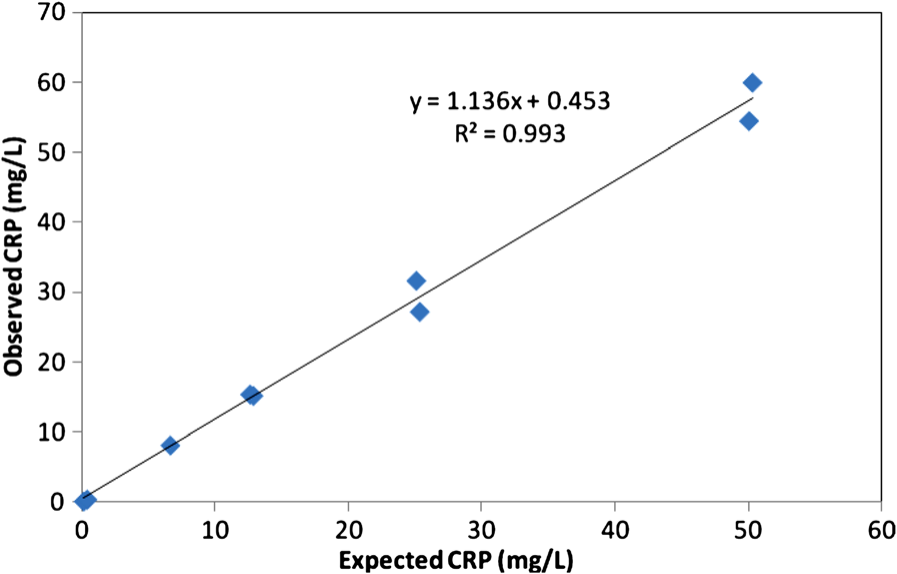
Accuracy of the CRP ELISA for determination of CRP in synovial fluid: recovery of spiked CRP from pooled synovial fluid.

**Figure 7:**
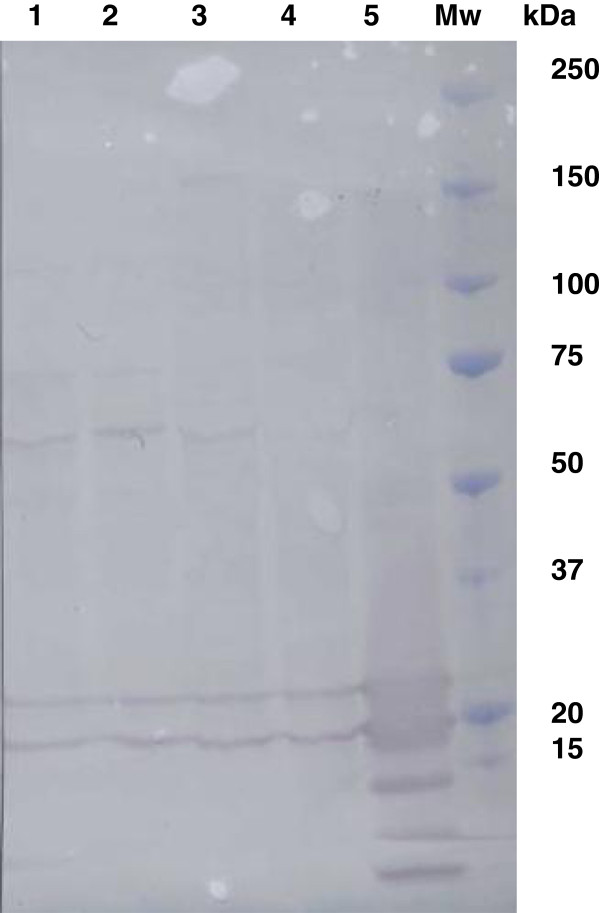
Western blot of canine synovial fluid (Tracks 1& 2), canine serum (tracks 3 & 4) purified canine CRP (track 5) and molecular weight markers (Mw).

Validation of the ELISA to measure CRP in synovial fluid showed an intra-assay co-efficient of variance (CV) of 8.9 +/− 7.9% (mean ± SD) for duplicate samples (n = 34). The precision profile based on the CVs of the duplicates (Figure 4) showed that higher CVs were given with low concentrations of CRP in synovial fluid. Inter-assay CVs were 19.4% at a mean concentration of 6.2 mg/L and 17.5% at a mean concentration of 49.1 mg/L (n = 5). The accuracy of the assay in synovial fluid, determined by calculating the recovery of CRP spiked into a pool of the fluid showed a recovery of 113% (Figure 6) and a correlation of R^2^ = 0.993 for expected against observed concentrations. The assay had a limit of detection of 0.05 mg/L, based on the concentration detectable at 3 standard deviations from the mean of the blank samples over 7 assays and allowing for the dilution of synovial fluid (1:200) used in the assay. The Western blot (Figure 7) confirmed the presence of CRP in synovial fluid with the double band known to be present in this species evident in both synovial fluid and serum samples.

**Figure 8:**
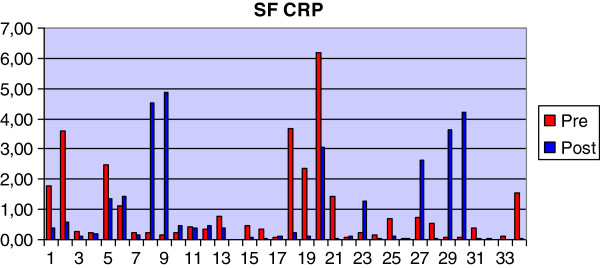
Graph showing pre- and post-treatment values for CRP concentration in SF.

Nineteen of 26 cases had a lower concentration of CRP in the post-treatment sample (Figure 8). Seven cases had an increase. The post- treatment CRP concentration in synovial fluid (0.17 mg/L; 0.05-0.49 mg/L) was significantly lower compared to the pre-treatment level (0.44 mg/L; 0.23-1.62 mg/L) (p < 0.05).

The concentrations of CRP in both the pre- and post-treatment synovial fluid samples were significantly greater than the levels (median 0.14 mg/L SD+/− 0.32 mg/L) measured in normal synovial fluid samples (p < 0.05).

There was no correlation between levels of CRP in matched serum and synovial fluid samples, neither in the pre-treatment nor post-treatment samples.

## Discussion

A 28-day course of Robenacoxib proved effective in reducing the lameness scores of dogs with stifle osteoarthritis secondary to failure of the cranial cruciate ligament. The reduction in lameness scores was statistically significant. It is accepted that a deficiency of the study was the use of a subjective assessment of lameness although all the lameness assessments were carried out by the same individual who was not aware of the initial score when re-evaluating the animal a month later. More objective measurements of lameness such as force plates and pressure mats were not available for this study. The use of activity monitors was not feasible since part of the case management involved restricted exercise during the course of the NSAID. The analgesic properties of NSAIDs for joint pain are well known and clinical improvement has been reported with many different compounds [37] Robenacoxib preferentially inhibits the COX-2 enzyme which is involved in the pathophysiology of joint pain and central sensitization [2]. It is possible that some of the clinical improvement was due to the restricted exercise regime used, although all of the dogs had already been placed on a controlled exercise plan by the referring veterinarian.

Although in 58% of cases the radiographic scores did not change, overall there was a statistically significant increase in the radiographic score over the period of the study indicating that the radiographic changes had progressed. This is to be expected since once initiated, OA is a gradually progressive disease and there is little evidence that NSAIDs can influence the structural pathological changes that occur within the joint. The radiographic score that was used in this study is very much biased towards assessing osteophyte formation and changes in bone opacity and the only study which has shown an effect of a NSAID on such bony changes is an experimental study in the dog [38,39]. These studies involved only small numbers of dog and the protocol meant that the NSAID was given from day one when the arthritis was initiated (by sectioning the cranial cruciate ligament), a situation very different from the clinical one, where the disease is generally well advanced before clinical signs are evident and treatment required.

The synovial fluid score based on total nucleated cell counts was not significantly affected by the course of Robenacoxib. Although it is generally accepted that the total nucleated cell count (neutrophils, lymphocytes and macrophages) does reflect the level of inflammation within the joint [40], there have been no studies validating the use of nucleated cell counts as an outcome measure in the treatment of osteoarthritis.

A major finding in this study was the statistically significant decrease in the concentration of CRP in synovial fluid following the month’s course of Robenacoxib. There was no statistically significant difference in the concentration of serum CRP between the pre- and post-treatment samples. This supports the view of Hurter et al. [33] who concluded that serum CRP was not a useful marker of OA in the dog. Our study is the first to have reported CRP concentrations in the synovial fluid of dogs and the results support the possibility that synovial fluid CRP is a useful biomarker of joint inflammation and a useful monitor of response to anti-inflammatory treatment. The concentrations of CRP detected in the synovial fluid were much lower than the blood levels. Although the concentrations of synovial fluid CRP in the OA dogs were statistically significantly higher than found in normal synovial fluid, in approximately a third of cases the levels fell within the normal range even though they still decreased after therapy. The measurement of these relatively low levels of CRP does require a very sensitive assay such as the ELISA used in this study. Immunoturbidimetric assays, used in previous reports [41,42] had a limit of detection of 1 mg/L and are thus not sensitive enough to provide meaningful results, whereas the ELISA used in our study had a lower limit of detection of 0.05 mg/L. The assay was validated for use in measuring this protein in synovial fluid and demonstrated equivalent precision to that for serum CRP while the accuracy of the assay was also acceptable with a recovery rate of 113% from spiked samples. Whilst the antibody used in the Western blot was not the same as used in the ELISA, the immune blot does confirm that CRP is present in canine synovial fluid.

It is possible that drugs used in inducing and maintaining general anaesthesia can cause alterations in the blood (and therefore perhaps also in synovial fluid) concentration of CRP in both humans [43-45] and dogs [46] although in all these studies the patients also underwent surgery and this is much more likely to be the stimulus for elevation of CRP levels. In our study, both pre- and post-treatment blood samples were collected from conscious dogs but the 2 synovial fluid samples were collected whilst the dogs were anaesthetized, within minutes of the dog losing consciousness and before any surgical stimulus. The fact that both synovial fluid samples were collected under general anaesthesia means that comparison of CRP concentrations in the pre-and post-treatment samples should not be compromised, even if the anaesthetic agents do have an effect since the same anaesthetic protocol was always used and any effect of the anaesthetic is likely to take several hours [46].

The origin of synovial CRP is not known. It could originate from the blood or be locally produced by the synovial membrane. There was no correlation between the concentrations of CRP in synovial fluid and matched serum sample suggesting that levels in the synovial fluid were not merely a reflection of the serum levels. However, it is possible that CRP in synovial fluid is solely a result of transfer from blood but that there is sufficient variation in the rate of transfer between dogs leading to the lack of correlation. Alternatively, blood derived CRP may be diluted to varying degrees by the synovial fluid. Arthritic joints tend to have much greater volumes of synovial fluid than normal joints, which can vary between cases, although even in normal joints synovial fluid concentrations of CRP tend to be lower than matched blood levels. Local production of CRP within the joint could also account for the lack of correlation of blood and synovial levels although to date, there have been no studies published to show local production of CRP within the joint although this has been shown with SAA in man [19,21], dog [34] and horse [47]. There have been several studies which have shown the local production of CRP in other inflamed tissues proving that the liver is not the only source although it is probably the major producer, particularly when the patient is suffering from a systemic illness. A study by Maekawa et al. [48] demonstrated a significantly elevated gene expression of CRP in human patients with gingivitis and periodontitis. The CRP gene was up-regulated in fibroblasts and endothelial cells rather than the epithelial cells of the oral mucosae. Another study demonstrated CRP gene expression in adipose tissue from human patients [49]. Adipose tissue is commonly found within the joint capsule and, in addition, the stifle joint contains the infra-patellar fat pad and this coupled with the fact that fibroblasts are the commonest cell found within the joint capsule means that it is highly likely that CRP is produced locally within the joint. Further studies, particularly determining CRP gene expression within the synovium and looking for synovial isotypes of CRP are required.

Robenacoxib has been shown to persist within inflamed tissues for much longer than it remains in the circulation [3]. This has lead to the concept of tissue selectivity [4] by which a drug concentrates in the inflamed tissues where it is required and has a prolonged anti-inflammatory effect. The fact that the half-life of the drug is reduced in the circulation means it is less likely to cause adverse effects on tissues such as the liver and kidney thus contributing to an improved safety profile. The fact that Robenacoxib appears to reduce synovial levels of CRP supports a local effect and the concept of tissue selectivity.

CRP production is mediated by the catabolic cytokines, particularly IL-6, TNFα and IL-1. It is known that PGE_2_ regulates the production of cytokines such is IL-6, IL-8, IL-11, macrophage colony stimulating factor and vascular endothelial growth factor [50-52], possibly involving the activation of the PGE_2_ receptors, EP2 and EP4 with an increase in cyclic AMP. By inhibiting PGE_2_ activity and thus cytokine production, NSAIDs will affect the acute phase response and also have other beneficial effects eg IL-6 is known to stimulate PGE_2_ production and matrix metalloproteinase production within the joint leading to articular cartilage damage, macrophage colony stimulating factor activates monocytes/macrophages to produce more catabolic cytokines and vascular endothelial growth factor plays an important role in angiogenesis and endothelial migration during the development of synovitis [53-56]. These effects would be additional benefits of using Robenacoxib (and other NSAIDs) in the treatment of osteoarthritis and help to explain why NSAIDs at the level of the joint may result in the slowing of disease progression [38,39]. We found no correlation between CRP concentrations (in blood or synovial fluid) and lameness or radiographical (pre-treatment) scores.

It is worth noting that in dogs 8, 9 and 30 where the NSAID was stopped prematurely, there was a marked increase in the concentration of synovial fluid CRP in the post-treatment sample (18, 33 and 49 fold increases respectively). This was significantly greater than for any other case where there had been an increase after treatment; in all these other cases, the animal was still receiving the NSAID when the post-treatment sample was taken. This observation raises the question as to whether the NSAID should be gradually withdrawn after a therapeutic course rather than finished abruptly although further studies are necessary to investigate this.

## Conclusions

Robenacoxib proved to be safe and effective in reducing lameness scores (p < 0.01) in dogs with failure of the cranial cruciate ligament and stifle OA. The drug did not significantly alter serum concentrations of CRP but did significantly reduce synovial fluid concentrations (p < 0.05), suggesting a local anti-inflammatory effect consistent with its property of being tissue selective. There was no correlation between matched serum and synovial fluid concentrations of CRP, raising the possibility of a local production of CRP within the joint, a possibility worthy of further investigation. CRP concentration within synovial fluid is a potential biomarker of articular inflammation and may prove useful in monitoring ant-inflammatory responses to therapeutic agents. Synovial fluid concentrations are much lower than those found in serum and their accurate measurement requires more sensitive assays (ELISA) than routinely used for serum.

## Methods

### Study protocol

A total of 34 dogs with OA secondary to failure of the cranial cruciate ligament were included in the study. All the dogs had to be free of other diseases and were not to have received any medication for the 15 days prior to presentation. The study was carried out in the Department of Veterinary Clinics at the University of Pisa and ethical permission for the study was granted by the committee for animal experimentation of the University of Pisa. The age, sex, breed, body weight, affected joint and duration of lameness were recorded for each dog. A full physical examination, including a detailed orthopaedic examination was carried out and a blood sample collected when the dog first presented. Each dog was anesthetized and a radiographic examination performed on both stifle joints and synovial fluid (from the affected joint) was collected. Because of the surgical waiting list at the clinic, it was normal practice to delay surgery for 2–3 weeks. In this study surgery was delayed for 4 weeks over which time each animal was given a course of Robenacoxib (TN.Onsior, Novartis Animal Health) at a dose rate of 1 mg/kg SID orally without food. All animals where then re-assessed at the end of the 4 week course, whilst still receiving the NSAID. Each animal had a second physical examination with a detailed orthopaedic examination and a second blood sample was collected, A further radiographic study was carried out and a second synovial fluid sample was collected whilst the dog was anaesthetized. Surgical treatment of the cruciate rupture was carried out at this time, after the samples had been collected. All the assessments were carried out by the same observer and the initial scores were not known to the observer when doing the re-assessments.

Whilst the dogs were receiving the NSAID, the owners were instructed to restrict their animal’s exercise to ten minutes walk on a lead, 3 times a day. This was similar to that which had already been instigated by the referring veterinarian. The owners were also asked to contact the clinic direct if any health problems arose during the study period.

### Lameness scoring

The dogs were examined standing and at the walk and the trot. A scoring system was used based on the severity of lameness using a modification of the method described by Peterson and Keefe [57] (Table 2).

**Table 2:** **Details of the lameness scoring scheme (Modified from Peterson and Keefe,**[57]**)**

**Score**	**Description**
0	No detectable lameness at a walk or trot, no detectable lateral weight shift whilst standing
1	No detectable lameness at a walk or trot and minor lateral weight shift whilst standing
2	Lameness at a trot
3	Lameness at a walk and trot
4	Non-weight bearing at a trot
5	Non-weight bearing at walk and standing

### Body condition scoring

Body condition scoring was assessed using the method described by Laflamme [58] ranging from 1 (emaciated) to 5 (obese).

### Radiographic scoring

A modification of previously described methods [35,59-61] was used. The presence and size of osteophytes, at 11 different anatomical sites were assessed on both a mediolateral and craniocaudal radiograph of the affected stifle joint; the sites were the lateral and medial trochlear ridges of the femur, lateral and medial condyles of the femur, proximal and distal poles of the patella, the fabellae, lateral/medial/caudal edges of the tibia and the intercondylar area of the femur. Based on the size of the osteophytes in millimetres, a score was given for each site (Table 3a). These scores were totaled and converted into a final score of 1–3 (Table 3b).

**Table 3:** Details of the radiographic scoring scheme

a) For each of 11 different sites, the size of osteophytes was measured in millimetres and a score was assigned:				
0	Absence of osteophytes			
1	Osteophytes smaller than 2 mm			
2	Osteophytes between 2 and 5 mm			
3	Osteophytes greater than 5 mm			
b)The osteophyte score for each site was totaled and then converted to a final score of 1-3				
From 0 to 10	Mild	1		
> 10 to 20	Moderate	2		
> 20	Severe	3		
c) The final osteophyte score, bone opacity score, joint effusion score and global score were summed to give the overall score (maximum possible 12)				
Parameter	Absent	Mild	Moderate	Severe
Osteophytes	0	1	2	3
↑ Bone opacity	0	1	2	3
Joint effusion	0	1	2	3
Global score	0	1	2	3
				**Total maximum score 12**

Joint effusion and an increase in radio-opacity of the distal femur (along the trochlear margin on the medio-lateral view) were also evaluated and scored as 0 (absent), 1 (mild), 2 (moderate) or 3 (severe). A global score, intended to represent a subjective impression of the overall disease severity, was also used, (0 normal joint, 1 mild changes, 2 moderate changes and 3 severe changes). The sum of all the recorded scores gave the total radiographic score for each joint, the maximum possible score being 12 (Table 3c).

### Synovial fluid scoring

Synovial fluid was examined macroscopically and microscopically and the scoring system used (from 0–3) was based on the automated nucleated cell count (Table 4). The total nucleated cell count gives an indication of the synovial inflammation [40] but has not been validated as a laboratory marker. Red blood cell counts in all the synovial fluid samples had to be less than 0.01 × 10^12^ cells per litre which is consistent with minimal blood contamination. This was important to ensure that significant amounts of serum CRP had not leaked into the joint and that nucleated cell counts accurately reflected synovial fluid numbers rather than blood contamination..

**Table 4:** Details of the synovial fluid scoring scheme (based on nucleated cell count)

**Category**	**Cell count**	**Score**
	**X10**^**6**^**/ml**	
Not increased	< 0.7	0
Slightly increased	0.7-1.5	1
Moderately increased	< 1.5-3.0	2
Severely increased	> 3.0	3

The total nucleated cell count was done on 0.5 ml of sample using the Hemat 8 (SEAC) analyzer. The differential cell count (neutrophils, lymphocytes and macrophages) was performed on a smear of synovial fluid after Romanowsky staining (Diff-Quik, Vetefarma). Before analysis the sample was treated with hyaluronidase (from bovine testes-Sigma, Aldrich) to reduce the viscosity (2 drops of a solution containing 150 U/ml of enzyme in PBS buffer for 0.25 ml of sample) [62].

### Measurement of C-reactive protein concentration

Synovial fluid was centrifuged immediately after collection in plain tubes at 1500 g for 3 minutes and the supernatant collected. All the serum and synovial fluid samples were stored at −80°C until the CRP concentrations were measured. CRP was measured using an ELISA [63] available commercially (Tridelta Development Limited, Ireland) and previously validated to measure CRP in canine serum [36] with further validation in this study (intra-and inter-assay coefficients of variance). The test was further validated for use with synovial fluid by assessment of precision and accuracy using this fluid as the sample matrix. Precision was determined by calculation of inter assay CV with three samples of synovial fluid assayed for CRP in 5 separate assays. Intra assay CV was calculated as the mean of the CV of duplicate samples of synovial fluid and also plotted as a precision profile. To determine accuracy of measurement of CRP in synovial fluid, purified canine CRP (Life Diagnostics, NJ, USA) was dispensed at 25 mg/L, 12.5 mg/L and 6.3 mg/L in a pool of synovial fluid with a low concentration of CRP. Following assay for CRP, the mean recovery of the CRP was calculated as a measure of the accuracy of the test.

The presence of CRP in synovial fluid was confirmed by antibody cross reactivity on a Western blot following sodium dodecyl sulphate polyacrylamide gel electrophoresis (SDS-PAGE) using established methods. The first antibody was a sheep antibody raised against purified canine CRP at a dilution of 1:200 and the second antibody was a rabbit anti- sheep IgG labelled with HRP (Abcam, Cambridge, UK) with the blot developed using an Opti-4CN kit (Biorad, Hemel Hempstead, UK). Samples were 2 synovial fluids and 2 sera with elevated concentrations of CRP as determined by ELISA and a purified CRP sample (Life Diagnostics, NJ, USA).

### Statistical analyses

Data obtained before and after treatment for the examined parameters (lameness score, radiographic score, synovial fluid score, CRP concentration in serum and synovial fluid) were compared by the Wilcoxon matched pair signed rank sum test for non-parametric values. Synovial fluid CRP concentrations before and after treatment were compared with normal values measured in a previous study, using the same assay (median = 0.14 mg/L +/− 0.32 mg/L), by the Wilcoxon matched pair signed rank sum test for non-parametric values. Correlation between the matched serum and synovial fluid CRP concentrations was measured by calculation of the Pearson correlation coefficient.

Eight cases were excluded from the statistical analyses for synovial fluid CRP, ten cases for the serum CRP analyses and 8 cases for the lameness, radiographic and synovial cell count analyses. The reasons for this were:

• With cases 8, 9 and 30 the treatment protocol was not completed since the administration of Robenacoxib was stopped prematurely (in case 8 at 3 weeks, in case 9 at 1 week and in case 30 at 10 days).

• With case 29 the labeling of the pre- and post-treatment samples was unclear.

• Case 28 had a very high concentration of CRP in the post-treatment serum sample and a few days after the second examination the dog showed signs of systemic infection, notably pyrexia, leucocytosis, vomiting and diarrhea which were not evident at the time of the physical examination and it was highly likely that the raised serum CRP was an early response to this systemic infection.

• Cases 14, 32 and 33 were not returned for the second examination after the owners declined surgery.

• The post-treatment serum samples for cases 18 and 19 were lost in the clinic.

## Abbreviations

APPs: Acute phase proteins; COX: Cycloxygenase; COX-2: Cycloxygenase 2; CRP: C-reactive protein; CV: Co-efficient of variance; ELISA: Enzyme linked immunosorbent assay; IL-1: Interleukin-1; IL-6: Interleukin-6; NSAID: Non-steroidal anti-inflammatory drug; OA: Osteoarthritis; SAA: Serum amyloid A; TNFα: Tumour necrosis factor alpha.

## Competing interests

Novartis Animal Health provided part-funding for one of the authors (SS) and contributed to the owners’ cost of the cruciate surgery as an incentive for participation in the study and they also provided the robenacoxib free of charge. They also paid for the purchase of laboratory consumables and test kits. They were not involved in designing the study protocol or in carrying out any of the data analyses. They made no contribution to the manuscript contents and had no influence on whether or where to publish.

## Authors’ contributions

DB designed the project and was responsible for its overall management and prepared the first and final drafts of the manuscript. He is funded by the University of Glasgow. PDE, MW and EMC carried out the C-reactive protein assays including the validations. PDE and MW are funded by the University of Glasgow, EMC by ReactivLab Ltd. VM and SS were involved in recruiting cases and carrying out the clinical evaluations. VM was funded by the University of Pisa and SS by both the University of Pisa and Novartis Animal Health (50% each). SS and PDE also helped in the preparation of the manuscript. AR carried out the statistical analyses. AR is funded by University of Pisa. All authors read, edited and approved the final manuscript. The University of Glasgow covered all costs of producing the manuscript.
